# Light‐Assisted 3D‐Printed Hydrogels for Antibacterial Applications

**DOI:** 10.1002/smsc.202400097

**Published:** 2024-05-23

**Authors:** Liwen Zhang, Naufal Kabir Ahamed Nasar, Xumin Huang, Chenyang Hu, Xuan Pang, Xuesi Chen, Ruirui Qiao, Thomas Paul Davis

**Affiliations:** ^1^ Australian Institute of Bioengineering & Nanotechnology The University of Queensland Brisbane QLD 4072 Australia; ^2^ Key Laboratory of Polymer Ecomaterials Changchun Institute of Applied Chemistry Chinese Academy of Sciences 5625 Renmin Street Changchun 130022 P. R. China

**Keywords:** 3D printing, antibacterial and tissue engineering, hydrogel

## Abstract

Light‐assisted 3D printing technology, which uses a light source to solidify a photopolymerizable prepolymer solution, has shown great potential in the development of antibacterial hydrogels with high‐resolution, specific features and functionalities. 3D‐printed hydrogels with customized structures and antibacterial functions are widely used in tissue engineering, regenerative medicine, wound healing, and implants to advance the modeling and treatment of diseases. In the current review, an overview of light‐assisted 3D printing technologies is first provided for the development of antibacterial hydrogels. Novel strategies involving the integration of inorganic nanomaterials, antibiotics, and functional polymers into 3D‐printed hydrogels for the enhancement of antibacterial effects are then discussed. Finally, the perspective of advanced design using artificial intelligence and machine learning is proposed, providing a comprehensive yet succinct examination of 3D‐printed hydrogels for antibacterial purposes.

## Graphical abstract

This review explores light‐assisted three‐dimensional (3D) printing technologies for fabricating natural and synthetic hydrogels with antibacterial properties. It highlights the integration of inorganic nanomaterials, antibiotics, and functional polymers into 3D‐printed hydrogels to improve antibacterial efficacy. Furthermore, this review discusses the prospects of artificial intelligence to advance the design and manufacture of 3D‐printed hydrogels.

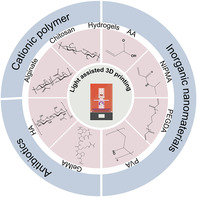

## 1 Introduction

Currently, ≈13 million patients globally face perilous bacterial infections each year. Particularly noteworthy is the estimation that during the COVID‐19 pandemic, up to 95% of pneumonia‐related deaths were attributed to secondary *Streptococcus pneumonia (S. pneumonia)* infections.^[^ ^1^ ^]^ Antibiotics have historically effectively treated bacterial ailments since Fleming discovered penicillin in 1928.^[^ ^2^ ^]^ However, the overuse and long‐term use of antibiotics have spurred the emergence of multidrug‐resistant bacteria, including superbugs.^[^ ^3^ ^]^ Therefore, it is highly demanded to develop antibiotic‐free strategies against antimicrobial resistance. In recent years, antibacterial biomaterials have been increasingly recognized as promising alternatives in combating bacterial infectious diseases for their antibiotic resistance, biocompatibility, as well as versatility in various biomedical products to prevent infections.^[^ ^4^ ^]^

Hydrogels, i.e., highly hydrated 3D porous polymer networks, have been extensively studied in antibacterial applications. By either integrating antibacterial agents (e.g., metal ions or metal‐based nanoparticles, antibiotics) or utilizing inherent antibacterial activity derived from polymeric structures, hydrogels have shown great promise as an alternative solution to traditional antibiotic treatments.^[^ ^5^ ^]^ Due to high water‐holding capacity, biocompatibility, and network structures, hydrogel effectively improves the loading and release of antibacterial agents and reduces the cytotoxic impact of antibacterial agents.^[^ ^6^ ^]^ Nevertheless, a significant obstacle in the conventional fabrication of hydrogels is the difficulty of shaping them in predesigned geometries.^[^ ^7^ ^]^

Over the last few decades, significant efforts have been devoted to developing hydrogels with intricate structures and functional properties using 3D printing techniques, facilitating the design of patient‐specific constructs with optimized antibacterial properties.^[^ ^8^ ^]^ Some 3D printing methods, including direct ink writing^[^ ^9^ ^]^ and fused deposition modeling (FDM),^[^ ^10^ ^]^ have been used in the fabrication of materials with antibacterial activities to produce customized medical devices with antibacterial properties, such as meniscus replacement,^[^ ^11^ ^]^ wound dressings,^[^ ^12^ ^]^ and osteochondral repair.^[^ ^13^ ^]^ Despite exciting advantages, certain 3D techniques with higher printing temperatures (>100 °C) often lead to the degradation of thermosensitive drugs and biological agents, such as proteins, RNA, and enzymes, resulting in a reduced antibacterial effect.^[^ ^14^ ^]^ In contrast, light‐assisted 3D printing methods utilize a light source, such as a laser or a projector, to selectively solidify liquid resins at room temperature, showing great promise in printing hydrogels with thermosensitive components.^[^ ^15^ ^]^ Moreover, light‐assisted 3D printing, like stereolithography (SLA) and digital light processing (DLP),^[^ ^16^ ^]^ offers superior spatial resolution, finer details, better pattern fidelity, and faster fabrication speeds,^[15a]^ enabling the fabrication of hydrogel structures with high resolution and designed geometries.

Despite previous review articles focusing on the topic of antibacterial hydrogels,^[^ ^5^, ^17^ ^]^ the scope of this article is to provide a comprehensive review of antibacterial hydrogels fabricated by current state‐of‐the‐art light‐assisted 3D printing technologies. We first present a detailed overview of the light‐assisted 3D printing strategies for the development of antibacterial hydrogels. The fundamental principles, advantages, and disadvantages of various light‐assisted 3D printing techniques are introduced. Second, we describe the progress in the development of natural and synthetic hydrogels using light‐assisted 3D printing techniques and highlight their antibacterial effects. Subsequently, we focus on various strategies for enhancing the antibacterial effects of 3D‐printed hydrogels by integrating 1) inorganic nanoparticles; 2) antibiotics; and 3) functional polymers. Future perspectives on novel artificial intelligence‐based additive manufacturing techniques are presented, aiming to underpin the potential of next‐generation antibacterial hydrogels for applications in tissue engineering and regenerative medicine fields.

## 2 Light‐Assisted 3D Printing Techniques for Antibacterial Materials

**Figure 1:**
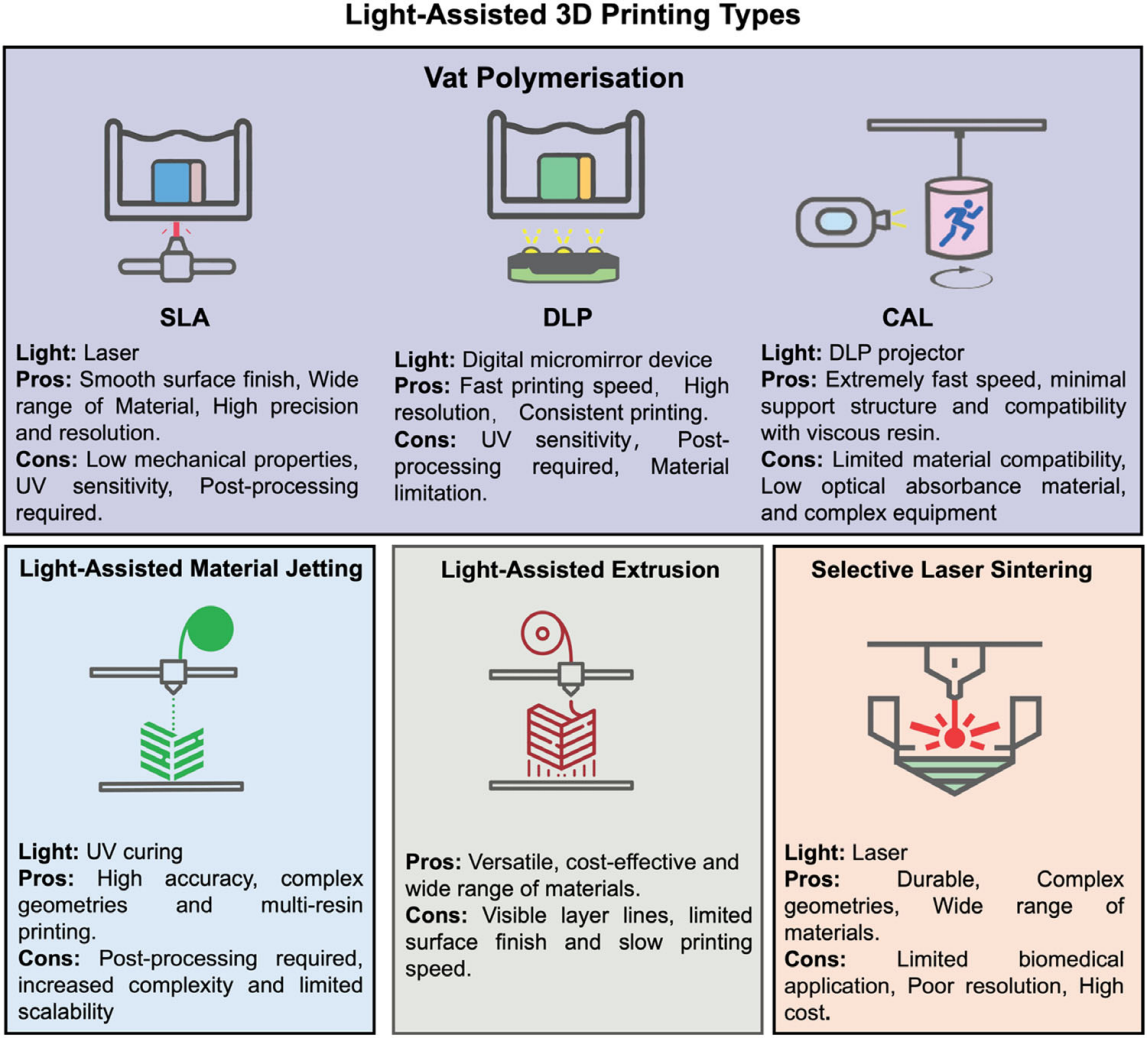
Summary of advantages and disadvantages of main light‐assisted 3D printing techniques.

3D printing, also known as additive manufacturing, is a process that builds 3D objects in a layer‐by‐layer manner from digital models designed by computer‐aided design (CAD) software. The CAD files are sliced into discrete layers, each printed on top of the other until the target object is fully formed.^[^ ^18^ ^]^ In contrast with conventional manufacturing techniques, 3D printing offers unparalleled design freedom, enabling the production of complex geometries and customized parts with ease. Its ability to rapidly prototype and manufacture low‐volume, high‐value items at a reduced cost makes it a valuable tool for various industries, from aerospace and automotive to healthcare and consumer goods.^[^ ^19^ ^]^ Its minimal material waste and on‐demand manufacturing capabilities also contribute to sustainability efforts and resource efficiency. In the last decades, numerous 3D printing techniques have been extensively explored to develop and fabricate materials, presenting innovative avenues for antibacterial functions. Particularly, light‐assisted 3D printing systems, like DLP and SLA, enable the production of objects with a spectrum of geometrical complexities through precise spatiotemporal control over localized photopolymerization of resins. An overview of the strengths and weaknesses of each light‐assisted 3D printing technique is presented in **Figure** **1**.

Furthermore, light‐assisted 3D printing techniques have been applied across various domains, including electronics, bioengineering, drug delivery, and antibacterial therapy. In the field of electronics, this technology enables the precise fabrication of intricate circuits and components directly onto diverse substrates, facilitating the development of compact, flexible electronic devices that are crucial for advancing wearable technologies and smart devices. In bioengineering, it plays a crucial role in producing customized, biocompatible implants and scaffolds with controlled microarchitectures, enhancing cellular interactions and tissue integration. Additionally, light‐assisted 3D printing contributes to the development of advanced drug delivery systems capable of dispensing medications in a controlled manner, adapted to the patient's specific physiological conditions. Moreover, this technology supports the production of objects with antibacterial properties, which are important for manufacturing medical devices and hospital equipment that minimize bacterial growth and mitigate infection risks.

### 2.1 Vat Photopolymerization

Vat photopolymerization is a general term for SLA and its related 3D printing process, where the curing of 3D constructs is induced by laser. This light‐assisted method, pioneered by Chuck Hill in the early 1980s, is rooted in traditional SLA principles. SLA utilizes a process where liquid resin undergoes solidification via a photopolymerization reaction, building up layer‐by‐layer to create 3D‐printed objects. The UV laser is used to trace each layer of the 3D object onto the surface of the liquid resin, initiating a photochemical reaction with the monomer resin and causing solidification.^[^ ^20^ ^]^ The process of SLA photopolymerization is conducted in a point‐by‐point fashion, where the solidification takes longer to create an object, but it does so with complex geometries, high resolution, and a smooth surface finish. SLA not only overcomes limitations of resolution and precision in traditional nozzle‐based 3D printing but also achieves a superior surface finish.^[^ ^21^ ^]^ Aside from the resins’ compatibility with various solvents, chemicals, and reagents, SLA is favored for its versatile material selection, thereby expanding the range of available printing materials. Despite their relatively slower printing speed, SLA 3D printers have become immensely popular for their ability to accurately produce precise parts with high fidelity in various photopolymer‐based materials, featuring fine details and a smooth surface finish.^[^ ^22^ ^]^

SLA 3D printing has been able to fabricate antibacterial materials using biocompatible composite materials. Vidakis et al. utilized a commercially available urethane dimethacrylate‐based resin conjugated with copper nanoparticles (CuNPs) for antibacterial applications. It was found that 2% CuNPs have an intensified zone of inhibition for both *Escherichia coli (E. coil)* and *Staphylococcus aureus (S. aureus)*, at 4.3 and 5.6 mm, respectively.^[^ ^23^ ^]^ Similarly, Marin and co‐workers used poly(methyl methacrylate, PMMA)‐based resin conjugated with ceramic powders to create antibacterial conjugate materials through SLA 3D printing. It was found that PMMA in conjugation with aluminum nitride demonstrated the most effective antibacterial effect against *E. coli*, with a 30–60% reduction in optical density.^[^ ^24^ ^]^

The DLP method originated in the 1980s when Texas Instruments developed the Digital Micromirror Device (DMD), initially developed for projection displays and is a critical component of DLP printing.^[^ ^25^ ^]^ DMDs consist of many micromirrors, each acting as individual pixels capable of being either illuminated or unilluminated in a binary system. DMDs in DLP printing can precisely control these microscopic mirrors to modulate light, generating precise patterns for each layer of a 3D object.^[^ ^26^ ^]^ While SLA relies on laser technology and sequential printing spot‐by‐spot, DLP utilizes the DMD to simultaneously project cross‐sectional images derived from the CAD models, resulting in enhanced printing speeds compared to spot‐by‐spot SLA 3D printing. Furthermore, there are similar features between DLP and SLA, where DLP and SLA 3D printing are all based on liquid resin containing monomer or oligomer photopolymers that are solidified to a solid object via light‐mediated polymerization.^[^ ^27^ ^]^ SLA‐ and DLP‐derived objects require postprocessing, including eliminating excess resin and UV curing for increased solidification.

The DLP 3D printing technique shares some advantages with SLA 3D printing. DLP technology is also proficient in generating intricate geometrics with exceptional printing speeds, high resolution, and smooth surface finish without any visible layer lines. DLP's versatile material selection allows for tailored solutions, each offering distinct physical characteristics to accommodate various applications, including soft robots, wearable devices,^[^ ^28^ ^]^ tissue engineering, complex tissue modeling,^[^ ^29^ ^]^ and drug delivery.^[^ ^30^ ^]^ For example, Du and co‐workers have used crosslinkable polydimethylsiloxane–thiourea‐based resin for DLP 3D printing.^[^ ^31^ ^]^ The resultant elastomeric parts were produced to have tunable mechanical characteristics and robust elasticity. As such, high‐resolution human hand models, lattices, and porous structures were fabricated, with potential applications in the rapid fabrication of medical devices. Additionally, Hone and co‐workers have used DLP technology to fabricate a fibroin‐based hydrogel for tissue engineering. To recapitulate cartilage tissue laden with chondrocytes, silk fibroin polymer combined with glycidyl methacrylate shows promise for applications in chondrogenesis and in vivo transplantation of engineered cartilage.^[^ ^32^ ^]^ Furthermore, DLP 3D printing has created intricate composite structures with graphene oxide (GO)^[^ ^33^ ^]^ and sliver nanoparticles^[^ ^34^ ^]^ for antibacterial applications.

Computed axial lithography (CAL) is an innovative light‐assisted 3D printing technique that aims to develop 3D‐printed objects in a single step without needing a layer‐by‐layer deposition.^[^ ^35^ ^]^ CAL represents the intersection between typical SLA and computed tomographic imaging. Photopolymers are illuminated from multiple angles along a single axis to form the 3D‐printed object.^[^ ^36^ ^]^ The volumetric polymerization occurs through the photosensitive resin in an enclosed vat, where 2D images are projected using a DLP projector, and the convergence from different angles produces a 3D‐printed object. The unique benefit of CAL is the ability to render 3D objects from high‐viscosity resin without support structures. Meanwhile, the time taken to print is reduced drastically compared to traditional layer‐by‐layer methods.^[^ ^37^ ^]^ Despite its promising capabilities, CAL is still in the early stages of development, focusing on refining the printing process and compatibility with different types of photopolymers. As such, CAL 3D printing has yet to be fully explored for its potential in biomedical applications.

### 2.2 Light‐Assisted Inkjet Printing

The material jetting technique, also known as Inkjet 3D printing, utilizes gravity, pressure, and another mechanism to expel the droplets of the bioink to the working platform, solidifying under specific wavelengths of light to fabricate 3D objects.^[^ ^38^ ^]^ The critical point is that each printed layer must be cured by applying an appropriate curing step between successive deposition cycles. In this regard, UV light is directed onto the droplets on the build platform in the Material Jetting machine for curing. This photopolymerization/photocuring process utilizes a specific wavelength of light to solidify monomers/oligomers from a liquid state into solid objects.^[^ ^39^ ^]^ Unlike the wavelengths used by other light‐assisted 3D printing techniques such as SLA and DLP, which are confined per the 3D printer manufacturers’ specifications, material jetting's light source has no wavelength restrictions, allowing for increased versatility.^[^ ^40^ ^]^ Material jetting 3D printing has benefits, including multimaterial integration, high resolution, smooth surface finish, and the ability to produce complex geometries with fine details. Material jetting 3D printing significantly reduces ink consumption by restricting ink ejection only to the intended deposition areas on the surface. Mugnaini et al. synthesized photocrosslinkable methacrylic pullulan, utilizing it as a bioink. The aqueous dispersions of methacrylated pullulan served as the substrate for inkjet printing.^[^ ^41^ ^]^ However, material jetting does have some limitations. It has a relatively slow printing speed and high price compared to other 3D printing techniques. Similarly, the choice of bioinks is limited to ones that process low viscosity, optimal degradation, and the ability to remain liquid during printing.^[^ ^42^ ^]^ Additionally, printed materials often exhibit lower mechanical strength and are prone to fracturing under mechanical stress compared to structures fabricated using traditional approaches. Particularly within the UV curing process, some materials with limited photostability may compromise their properties upon prolonged exposure to ambient light. The ongoing development of new ink materials holds significant promise in overcoming these persistent limitations. Although there are some shortcomings, material jetting printing has been successfully employed in antibacterial, tissue engineering, and pharmaceutical agents.^[^ ^43^ ^]^

MultiJet 3D printing is another nozzle‐based 3D printing technology renowned for its precision and versatility. Similar to Inkjet 3D printing, this technique deposits photopolymer droplets layer‐by‐layer to create a 3D object. The distinguishing characteristic of MultiJet 3D printing is its capability to use multiple materials simultaneously, enabling the fabrication of complex structures with diverse properties within a single print job. The MultiJet printer utilizes multiple UV‐curable liquid plastics that are thermosensitive or photosensitive, which are jetted through a multiextruder to print multimaterial objects simultaneously.^[^ ^44^ ^]^ The deposition of multiple materials as the core function of MultiJet printing allows for highly accurate and finely designed objects with multiple components to be fabricated, which have a better surface finish than material extrusion methods.^[^ ^45^ ^]^ MultiJet printing allows for certain advantages, such as exceptional precision and ease of use when incorporating multiple photopolymers. Multimaterial 3D printing has been applied to fabricate cardiac microphysiological devices to mimic native cardiac tissues in vitro.^[^ ^46^ ^]^ Using six different bioinks and multistep lithography allowed for the fabrication of cardiac devices that can reconstruct tissue mechanics. As such, this method of 3D printing has been applied in many biomedical settings such as antibacterial and fluidic systems,^[^ ^47^ ^]^ tissue engineering,^[^ ^48^ ^]^ and drug delivery.^[^ ^49^ ^]^ For example, He et al. utilized inkjet 3D technology to synthesize materials to combat biofilm formation due to bacterial colonization. It was found that poly‐tricyclodeadimethanol diacrylate‐based 3D‐printed objects had a 99% reduction in biofilm formation from *P.C aeruginosa* compared to traditional silicon‐based prosthetics.^[^ ^50^ ^]^ Similarly, Adarkwa and co‐workers, using a hybrid inkjet 3D printing system, synthesized 3D‐printed coating using poly(lactic*‐co*‐glycolic) acid (PLGA) and polycaprolactone (PCL) coupled with amorphous calcium phosphate and vancomycin. The release kinetics of the material were shown to have high bioactivity against *S. aureus*, as demonstrated by the zone of inhibition, where the concentration of vancomycin released was above the inhibitory and bactericidal concentrations.^[^ ^51^ ^]^

### 2.3 Light‐Assisted Extrusion Printing

Material extrusion‐based 3D printing is an additive manufacturing technique that uses a nozzle to continuously extrude the ink, depositing material layer‐by‐layer to yield 3D structures.^[^ ^52^ ^]^ One of the extrusion‐based 3D printing techniques is FDM, which has become a standard tool for manufacturing businesses, prototyping, jewellery, science, and education design on both desktop and industrial scales. FDM technology employs thermal energy to liquefy the filament or wire and extrude materials through a nozzle.^[^ ^53^ ^]^ The liquified biomaterial harnesses gravity, fluidics, and other pressure‐driven mechanisms to expel the droplets to fabricate a range of constructs. In this approach, the printing nozzle traverses the *X* and *Y* axes to generate each layer, while the vertical motion in the *Z* axis permits incremental layer‐by‐layer material deposition.^[^ ^54^ ^]^ The prominent advantages of FDM encompass its cost‐efficiency, user‐friendliness, versatility, and capacity to fabricate intricate structures employing a variety of polymer filaments.^[^ ^55^ ^]^ Specifically, FDM is known for its process simplicity, where the thermosensitive filament is melted and then solidified at ambient temperature, contributing to its cost‐effectiveness and making it a viable option for various applications. This 3D printing technique allows for well‐controlled and precise geometries and structure architecture.

However, the limitations of FDM printing are its slow printing speed, the presence of layer lines, and reduced resolution, which are the side effects of using the printing nozzles.^[^ ^56^ ^]^ Meanwhile, the reliance on a nozzle‐based continuous printing system constrains the production of high‐resolution scaffolds, where the size of nozzles, generally from 0.4 to 0.6 mm, results in layer lines and restricted resolution.^[^ ^57^ ^]^ Moreover, the high temperature required to melt certain materials leads to compromises in material quality. The high temperature of the nozzle also possibly leads to the loss of control of extrusion during the nozzle movement, resulting in the stringing phenomenon. FDM 3D‐printed objects need additional postprocessing operations to improve their appearance and strength, such as sanding, polishing, vapor smoothing, and hydrodipping with customized patterns.^[^ ^58^ ^]^ These limitations constrain the potential applicability of FDM in fabricating antibacterial materials, particularly for biomaterials necessitating robust mechanical strength. However, the FDM printing technique still exhibits its potential in creating antibacterial materials. Nyabadza et al. demonstrated increased antibacterial efficacy against *E. coli* using 3D‐printed polylactic acid (PLA) objects coated with CuNPs, exhibiting a 12.33 mm zone of inhibition.^[^ ^59^ ^]^ Bayraktar et al. also utilized PLA filament embedded with silver nanowires to fabricate antibacterial materials, showcasing antibacterial activity against *E. coli* and *S. aureus*, with a 100% killing rate for both pathogens after 2 h.^[^ ^60^ ^]^ These studies underscore the potential of FDM printing in combating bacterial contamination, offering versatile applications in healthcare, food packaging, and beyond.

With the development of material extrusion techniques, light‐assisted extrusion printing offers a strategy to address the limitations of high temperatures in traditional extrusion printing. The light can be integrated into any stage of extrusion printing: during printing,^[^ ^61^ ^]^ postprinting,^[^ ^62^ ^]^ or after the deposition of each extrusion layer.^[^ ^63^ ^]^ For example, Ouyang et al. developed a generalizable in situ‐crosslinking strategy in which the light was introduced immediately before the deposition to achieve high encapsulated cell viability (≈95%).^[^ ^61^ ^]^

### 2.4 Selective Laser Sintering

Selective laser sintering (SLS) 3D printing utilizes a high‐power laser to heat and fuse powdered materials layer‐by‐layer. In SLS 3D printing, a layer of powdered material is spread across the build platform and then selectively fused by a laser according to the CAD model.^[^ ^64^ ^]^ This process is repeated layer by layer until the object is fully formed, allowing for the creation of complex and precise 3D structures.^[^ ^65^ ^]^ The major benefit of this 3D printing technique is the enhanced strength and durability of the material, which can be applied in the automotive^[^ ^66^ ^]^ and healthcare industries.^[^ ^67^ ^]^ However, a significant limitation of SLS 3D printing is its lower resolution compared to other light‐assisted 3D printing techniques such as SLA and DLP, resulting in less detailed and defined structure. Due to the higher temperature used in the printing process, the application of SLS technology in producing antibacterial materials remains challenging.

## 3 Light‐Assisted 3D‐Printed Hydrogels

Hydrogels are widely recognized for their significance and versatility in biomedical applications. Hydrogels are polymeric network structures with inherent hydrophilic properties, derived from natural and synthetic polymers. Their defining features encompassed the ability to uptake water many times their dry weight and insolubility in water.^[^ ^68^ ^]^ Typically, the crosslinking of the polymer network involves the reaction between one or more monomers, where the number of monomer and functional groups attached to the polymer backbone determines the degree of crosslinking. Currently, developing antibacterial hydrogels has become a main focus of biomedical research. Numerous cutting‐edge antibacterial hydrogels have been created, each with its exceptional characteristics, such as high water swellability, elevated oxygen permeability, enhanced biocompatibility, facile drug loading and release, and structural diversity. However, in numerous applications, the capacity to shape hydrogels into intricate 3D structures is indispensable. For instance, when hydrogels are regarded as a tissue scaffold, internal channels with high liquid flow permeability are necessary to sustain cell viability. Therefore, the versatility of light‐assisted 3D printing technology in creating complex structures and superior spatial resolution makes it a revolutionary tool for fabricating hydrogel‐based scaffolds for tissue engineering and antibacterial application. Moreover, light‐activation mechanisms in 3D‐printing were used in control structures of hydrogels from the millimeter scale to the submicrometer scale.

Hydrogels can be classified into natural and synthetic categories. Natural hydrogels typically consist of polymers derived from biological sources such as collagen, gelatine, hyaluronic acid (HA), and alginate.^[^ ^69^ ^]^ These hydrogels possess excellent biocompatibility, enabling their integration into biological systems and minimizing the risk of adverse reactions.^[^ ^70^ ^]^ Applications in complex scenarios are limited due to insufficient mechanical strength.^[^ ^71^ ^]^ In contrast, synthetic hydrogels, composed of polymers such as poly(ethylene glycol) (PEG),^[^ ^72^ ^]^ poly(acrylamide) (PAM),^[^ ^73^ ^]^ or PCL,^[^ ^74^ ^]^ have robust mechanical properties.^[^ ^75^ ^]^ However, synthetic hydrogels exhibit limited biocompatibility and generate degradation byproducts that are not found in nature.^[^ ^76^ ^]^ Despite their respective advantages and disadvantages, both natural and synthetic hydrogels are widely employed in light‐assisted 3D printing to create antibacterial hydrogels (**Table** **1**).

**Table 1:** Summary of light‐assisted 3D‐printed hydrogels and their properties.

Hydrogels	Light‐assisted 3D printing	Properties	Refs.
GelMAa)	SLA, DLP, CALb)	Easy antibiotic loading	[37, 163, 164]
Collagen	SLA, DLP	Biocompatible antibacterial effect against bacteria	[165, 166]
HA	SLA, DLP	Prolonged antibacterial activity with wound‐healing properties	[167, 168, 169]
Alginate	SLA, DLP	Potent antibacterial effect against bacteria	[170, 171]
Chitosan	SLA, DLP	Antibacterial Scaffold for bone tissue engineering	[172, 173]
Acrylic acid	SLA, DLP	Self‐healing hydrogel with antibacterial effect	[112, 174]
PEGDAc)	SLA, DLP	Stimuli‐responsive and antibacterial	[175, 176]
Poly(vinyl alcohol)	DLP	Strong antibacterial effect and self‐healing	[123, 177]

a) GelMA, gelatine methacryloyl.

b) SLA, *stereolithography*; DLP, digital light processing; CAL, computed axial lithography.

c) PEGDA, polyethylene glycol diacrylate.

### 3.1 Natural Hydrogels

Natural hydrogels are generally referred to as biopolymers derived from humans, animals, plants, and bacteria. Natural hydrogels can be divided into three groups according to their chemical structure difference: polysaccharides, glycosaminoglycans (GAGs), and polypeptides/proteins.^[^ ^77^ ^]^ These naturally derived materials can form a natural extracellular matrix (ECM) with the assistance of various proteins, leading to excellent biocompatibility and cell affinity.^[^ ^78^ ^]^

#### 3.1.1 Polysaccharides‐Based 3D‐Printed Hydrogels

In the last decades, various polysaccharides have been successfully employed to fabricate functional natural hydrogels, including but not limited to alginate,^[^ ^79^ ^]^ methylcellulose,^[^ ^80^ ^]^ and chitosan.^[^ ^81^ ^]^ Among them, chitosan and alginate have been approved by the U.S. Food and Drug Administration due to superior safety and biocompatibility. Chitosan is one type of cationic polyelectrolyte composed of d‐galactose and *N*‐acetyl‐d‐glucosamine. Amine groups make chitosan more easily dissolved in acidic solutions and provide higher chelation affinity to metal cations, especially antibacterial cation metals.^[^ ^82^ ^]^ Srikanthan et al. utilized inkjet printing to fabricate hydrogel composed of chitosan and polyethylene oxide (*M*_w_ = 1 000 000 g mol^−1^).^[^ ^83^ ^]^ The 3D printing parameters displayed a printing speed of 17–29 mm s^−1^, extrusion pressure between 21 and 24 kPa, and printing times of 0.3–0.5 h. These 3D‐printed scaffolds exhibited meshy structures capable of incorporating antibacterial zinc oxide nanoparticles. Notably, the hydrogel was very sensitive in acidic pH conditions to release zinc oxide nanoparticles for inhibiting the growth of Gram‐positive (*S. aureus*) and Gram‐negative (*E. coli*) bacteria. As 3D printing resolutions are highly dependent on the size of the printer nozzle and the postcuring process, it is difficult to manufacture materials with precise control and high resolution. The limitations of chitosan‐based materials are low mechanical resistance, poor control over pore size, and low solubility in neutral pH.^[^ ^84^ ^]^

**Figure 2:**
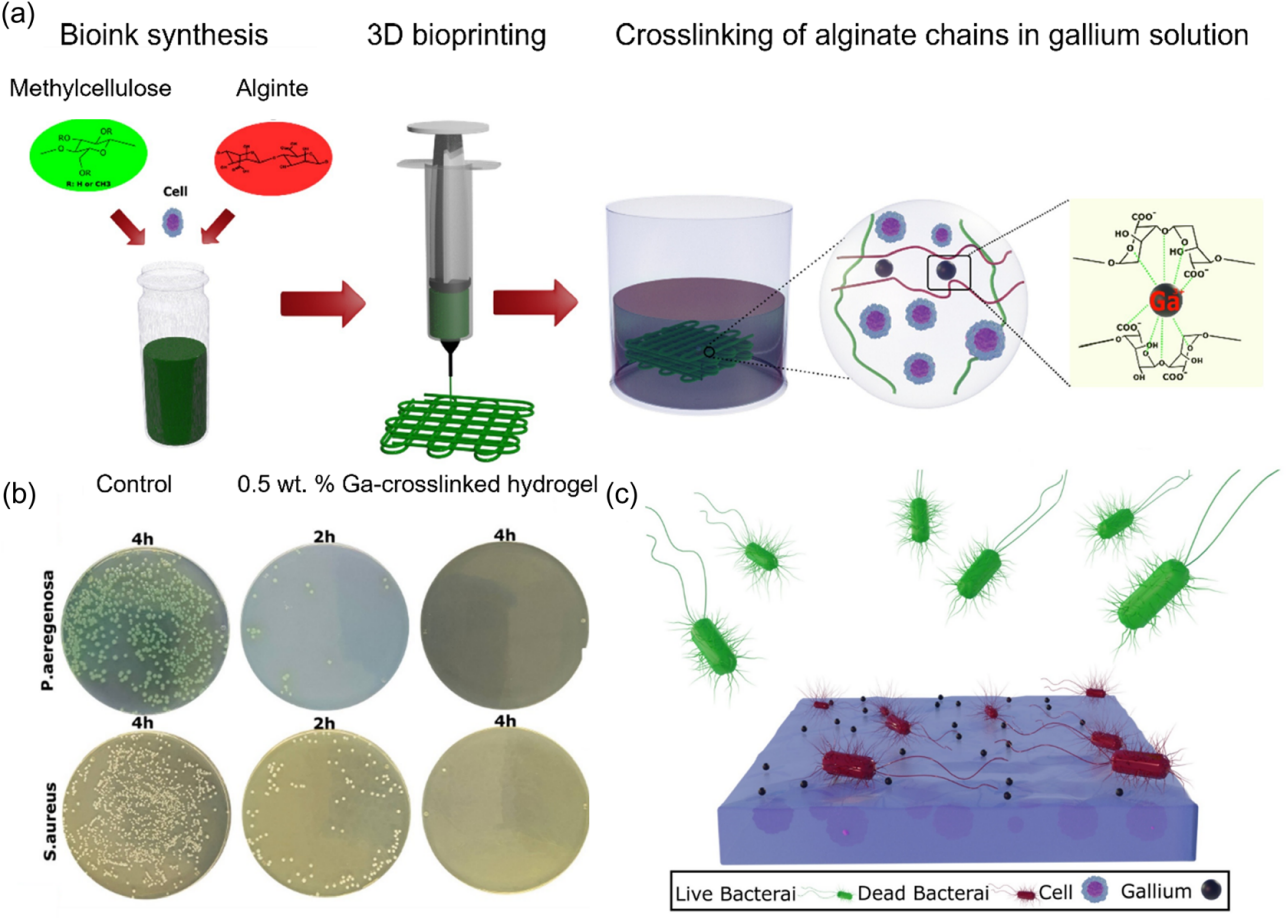
a) Schematic illustration of the 3D printing process of methylcellulose and alginate hydrogel composites. b) Surface antibacterial activity of the hydrogel toward *S. aureus* and *P. aeruginosa* bacteria. Surviving *S. aureus* and *P. aeruginosa* colonies on agar plates after contact with 0.5 wt% gallium‐crosslinked hydrogels. The gallium‐crosslinked hydrogel kills almost all bacteria in contact with the hydrogel surface. c) Schematic illustration of the antibacterial activity of gallium‐crosslinked hydrogel. Accordingly, the antibacterial activity of the hydrogel stems from the presence of gallium cations on the hydrogel surface. Reproduced with permission.^[^ ^87^ ^]^ Copyright 2021, Elsevier.

Alginate is a water‐soluble linear polysaccharide extracted from brown algae or bacteria.^[^ ^85^ ^]^ The gelation mechanism of alginate is different from chitosan, in which alginate self‐assembles into acidic gels in a lower pH environment. A distinctive feature of alginate is its biodegradability and decreased mechanical properties over time, making them ideal candidates for tissue engineering and regenerative medicine. Meanwhile, alginate‐based materials can cooperatively bind with metal cations, including Ca^2+^, Mg^2+^, Sr^2+^, Ba^2+^, or Al^3+^^[^ ^86^ ^]^ providing a new opportunity for antibacterial hydrogel composites. For instance, Hadi et al. employed inkjet printing to manufacture cell‐laden hydrogel composites containing alginate and methylcellulose (**Figure** **2**).^[^ ^87^ ^]^ In this study, gallium chloride not only induces the gelation of alginate but also endows the hydrogel composites with superior antibacterial effects. The results showed that the gallium‐crosslinked bioink exhibited potent antibacterial activity toward both *S. aureus* and *P. aeruginosa* bacteria, with a growth rate of 99.99%.

#### 3.1.2 GAG‐Based 3D‐Printed Hydrogels

GAG‐based hydrogels are biomaterials composed of GAGs, long unbranched polysaccharides with repeating disaccharide units. GAGs have several species, including HA, chondroitin sulfate, and heparin.^[^ ^88^ ^]^ HA is one of the most representative anionic GAGs, composed of d‐galactose and *N*‐acetyl‐d‐glucosamine and commonly found in various connective and support tissues.^[^ ^89^ ^]^ HA has unique properties, such as viscoelasticity similar to in vivo structures, biocompatibility, and increased water retention capacity.^[^ ^90^ ^]^ As a critical component of the ECM, HA also possesses essential functions in proliferation, migration, and tissue hydration. HA has been widely applied in biomedical applications, such as drug delivery,^[^ ^91^ ^]^ tissue regeneration,^[^ ^92^ ^]^ osteoarthritis treatments,^[^ ^93^ ^]^ and so on. Additionally, HA‐based hydrogels have become a popular topic in biomedicine. These hydrogels provide a supportive, robust matrix that mimics a natural tissue environment and can be safely degraded by hyaluronidases, allowing for absorption and metabolism within living systems without adverse effects.

**Figure 3:**
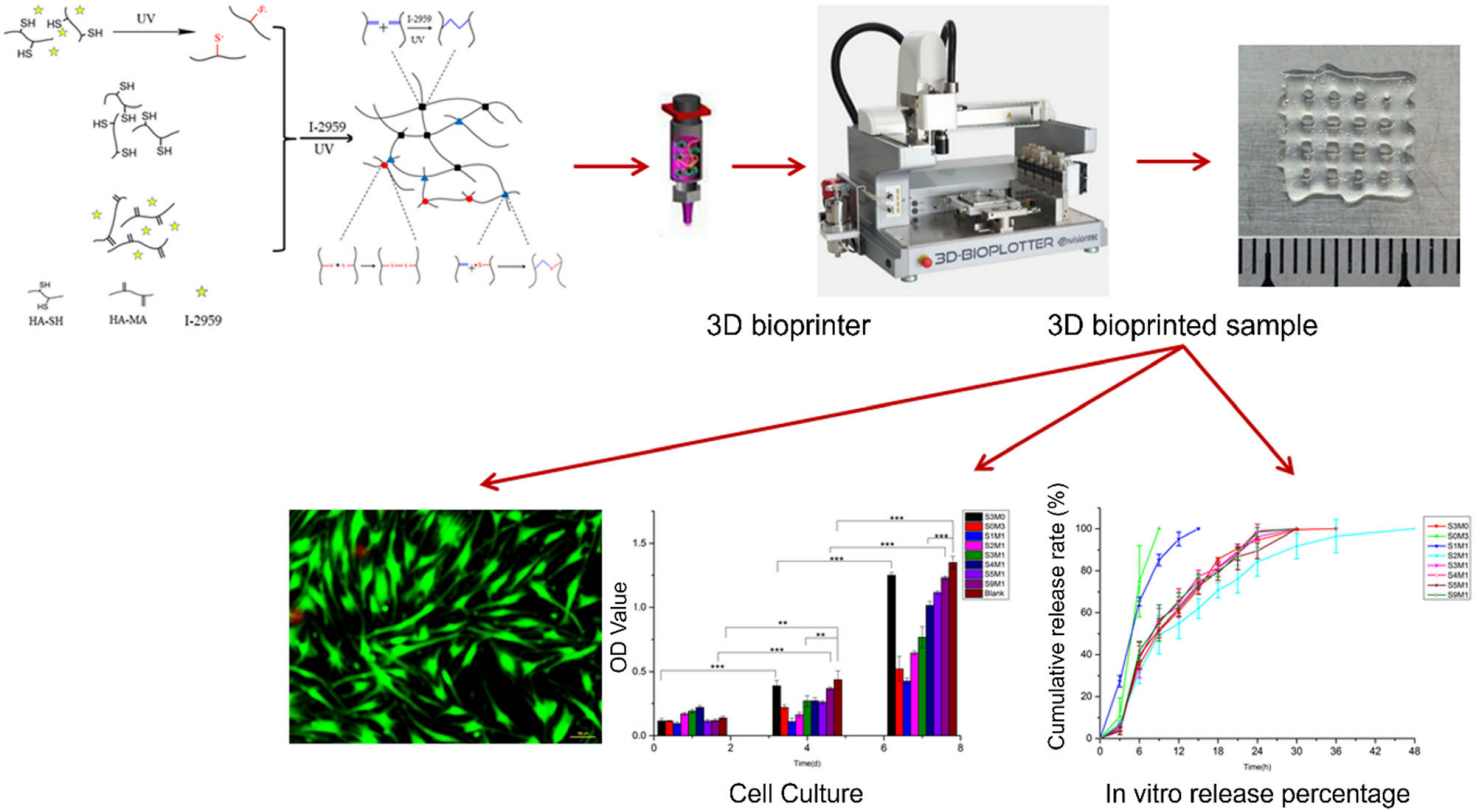
The schematic illustrates the fabrication process of double‐crosslinked materials, incorporating a UV‐crosslinker, HAMA, and click‐reaction crosslinked DTP‐modified HA. In vitro cytotoxicity testing revealed the potential of the drug‐loaded HA hydrogel for wound dressings. Reproduced with permission.^[^ ^95^ ^]^ Copyright 2021, MDPI.

More importantly, HA can be regarded as a photocrosslinker to generate stable hydrogels when exposed to light of specific wavelengths. A standard method to modify HA involves methacrylate substitution, generating hyaluronic acid methacryloyl (HAMA) with multiple chemical crosslinking sites (acrylamide groups).^[^ ^94^ ^]^ This chemical‐modified HA can be used as a bioink in light‐assisted 3D printing processes (SLA and DLP), where the hydrogel can be selectively solidified layer‐by‐layer to create complex structures. Interestingly, HAMA, a natural ECM component with anti‐inflammatory effects promoting cell adhesion and proliferation, has become an exciting candidate for tissue engineering and regenerative medicine. Si et al. developed a double‐crosslinked HA bioink containing UV‐crosslinker HAMA and click‐reaction crosslinked 3,3′‐dithiobis (propionyl hydrazide) (DTP)‐modified HA (**Figure** **3**).^[^ ^95^ ^]^ This bioink was successfully employed in the 3D extrusion printing process, and UV light (365 nm) was applied to crosslink the samples to obtain the final product. By mixing them at different ratios of two crosslinkers, the hydrogels exhibited controllable degradation rates and high swelling capacities, making them suitable for wound healing applications by incorporating the antibacterial drug nafcillin. However, the HA‐based hydrogels are generally brittle or have weak mechanical strength. Although HA is generally well tolerated by the body, there is still a risk of immunogenicity.

#### 3.1.3 Polypeptide/Protein‐Based 3D‐Printed Hydrogels

There are numerous natural polypeptides and proteins adopted into light‐assisted 3D printing, including but not limited to gelatine,^[^ ^96^ ^]^ fibrin,^[^ ^97^ ^]^ and collagen.^[^ ^98^ ^]^ Gelatine methacryloyl (GelMA), a chemically modified form of gelatine via a methacrylate process pioneered by Van Den Bulcke and co‐workers,^[^ ^99^ ^]^ is a notable example. This modification enables GelMA to become a crosslinkable polymeric monomer, allowing photoinitiated polymerization reactions to form stable polymer networks. GelMA, as a popular bioink, has been applied in light‐assisted 3D printing techniques to fabricate biomedical hydrogels with high‐resolution and complicated structures owing to their inherent biocompatibility, biodegradability, low immunogenicity, and overall printability.^[^ ^100^ ^]^ Interestingly, the mechanical properties of 3D‐printed GelMA hydrogels are directly influenced by the degree of substitution, as methacryloyl groups replace reactive hydroxyl and amine groups on gelatine amino acid residues.^[^ ^101^ ^]^ A higher degree of substitution typically increases crosslinking density, enhancing the hydrogel network's stiffness and strength to cater to various application requirements.

**Figure 4:**
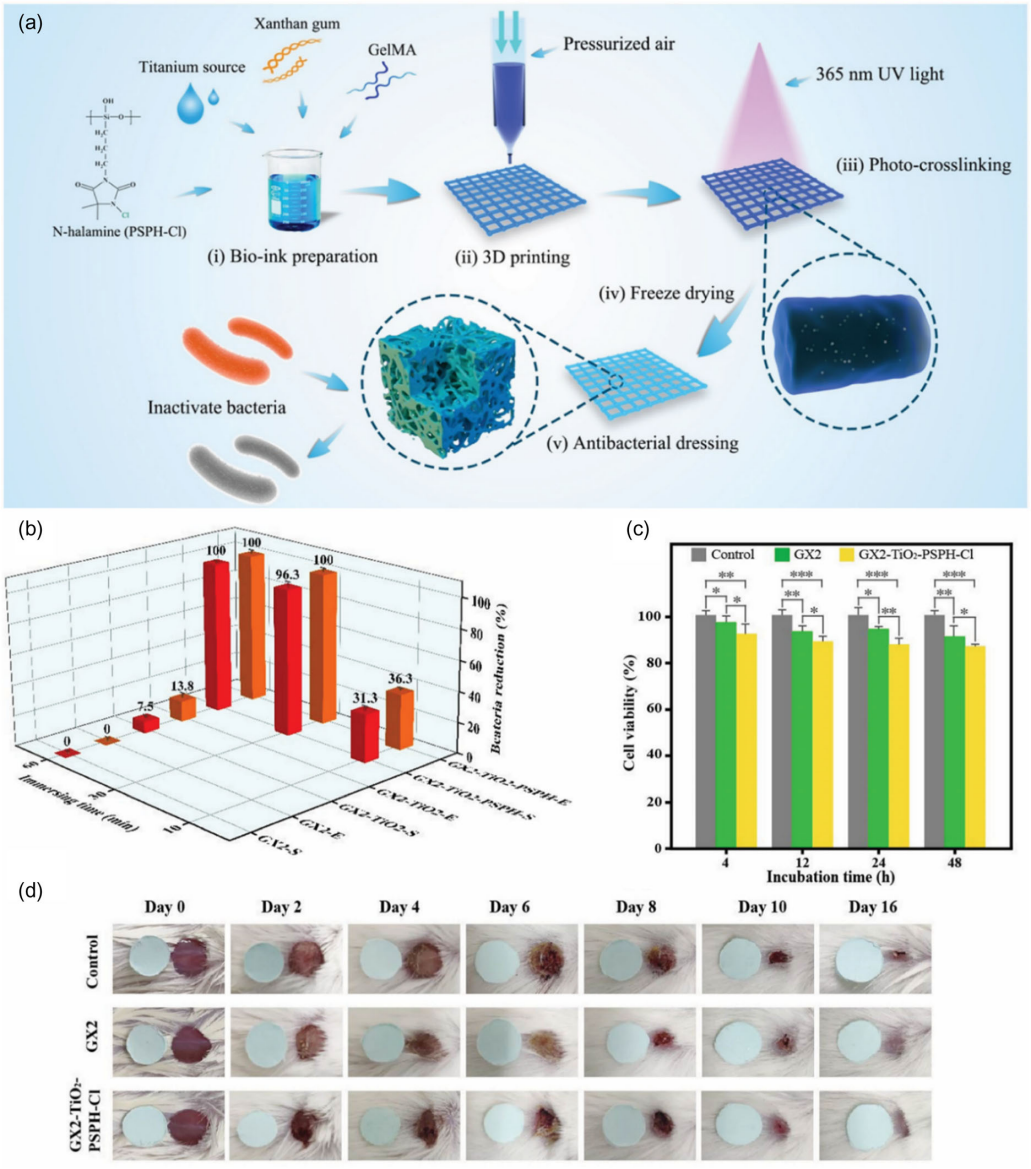
a) Schematic illustration of the fabrication of antibacterial 3D printing dressings. b) Biocidal results of GX2, GX2‐TiO_2_, and GX2‐TiO_2_‐PSPH‐Cl dressings with shake flask test against *S. aureus* and *E. coli* O157:H7. c) Cell viability of GX2 and GX2‐TiO_2_‐PSPH‐Cl dressings after 4, 12, 24, and 48 h incubation (*p ≤ 0.05, **p ≤ 0.01, and ***p ≤ 0.001). d) Representative photographs of wounds treated with gauze, GX2, and GX2‐TiO_2_‐PSPH‐Cl dressings at 0–16 days. Reproduced with permission.^[^ ^104^ ^]^ Copyright 2021, Elsevier.

3D‐printed GelMA hydrogels are a promising candidate for antibacterial applications. Vigata and co‐workers have developed a GelMA‐based local drug delivery system, aiming to increase the effectiveness of antibiotics at the site of infection and reduce the systematic side effects of antibiotics. GelMA hydrogel discs were loaded with Cefazolin with a loading efficiency of 99%. Additionally, the effectiveness and bioactivity of Cefazolin were tested against *S. aureus*, demonstrating a dose‐dependent manner of antibiotic activity and growth inhibition.^[^ ^102^ ^]^ Visscher and co‐workers integrated 3D printing with conventional salt‐leaching techniques to fabricate a novel scaffold for loading and delivering antibiotics.^[^ ^103^ ^]^ This scaffold comprised PCL for antibiotic loading, specifically Cefazolin, with a surface‐coated GelMA layer for prolonged antibiotic release. The results indicated that Cefazolin release was augmented in the 3D‐printed scaffolds and sustained for up to 3 days, exhibiting efficacy against *S. aureus*. It was found that 3D‐printed scaffolds containing the antibiotics had minimal reduction in the viability of fibroblast cells, indicating its excellent biocompatibility. Furthermore, Yang et al. printed hydrogel by combining GelMA and Xanthan gum (**Figure** **4**).^[^ ^104^ ^]^ In addition to antibiotics, *N*‐halamines with potent biocidal properties were introduced as the primary antibacterial component in the 3D‐printed hydrogels. Irradiation with a 365 nm UV light (10 W) was utilized to finalize the products. The 3D‐printed hydrogels demonstrated antibacterial solid activity, achieving complete inactivation of *E. coil* O157:H7 and *S. aureus* after 60 min of culture. Moreover, biofilm tests revealed the hydrogels’ ability to inhibit bacterial biofilm formation. These dressings exhibited excellent biocompatibility, and in vivo experiments in a mouse model demonstrated significant acceleration of wound healing, underscoring their potential as promising candidates for wound treatment.

Collagen, derived from different animal sources, is a widely used natural polypeptide in synthesizing hydrogels and other biomaterials.^[^ ^105^ ^]^ Approximately 28 types of collagens have different tissue distributions, functions, and cell identities. Type 1 collagen is the most prevalent, which consists of three alpha helices forming an overall helical structure. Like other naturally derived polymers in hydrogels, collagen offers advantages such as enhanced biocompatibility, reduced toxicity, and lower immunogenicity.^[^ ^98^ ^]^ Collagen‐based hydrogels have emerged as favorable environments for cell adhesion, proliferation, and tissue regeneration research, given collagen's structural resemblance to the native ECM.^[^ ^106^ ^]^ Moreover, collagen‐based hydrogels are a great candidate for wound healing and anti‐infection by providing structural support, promoting cellular migration, and facilitating tissue regeneration. Collagen as ECM regulates various stages of the healing process, ultimately contributing to effective wound closure and tissue repair. 3D printing is becoming a popular method for manufacturing collagen‐based hydrogels with precise resolution and customizable shapes.^[^ ^107^ ^]^ It is worth noting that 3D printing of collagen relies heavily on maintaining precise temperature conditions to obtain optimal printability. Collagen‐based inks undergo a thermoreversible gelation process, where increasing the temperature to 37 °C induces gelation while reducing the temperature leads to melting.^[^ ^108^ ^]^ Therefore, light‐assisted 3D printing techniques present an excellent option for fabricating collagen‐based materials, as they typically involve minimal heat generation during printing. Municoy and co‐workers developed 3D‐printed collagen scaffolds through in situ synthesizing sliver nanoparticles (AgNPs) by UV irradiation.^[^ ^109^ ^]^ Compared with AgNPs alone, collagen materials containing Ag exhibited enhanced antimicrobial activity against two bacterial strains, *E. coli* and *S. aureus*, possibly attributed to collagen's inherent hydration, which enhances the diffusion of silver ions.

### 3.2 Synthetic Hydrogels

Synthetic hydrogels consist of 3D swelling networks formed by the covalent or ionic crosslinking of hydrophilic homopolymers or copolymers.^[^ ^75^ ^]^ The polymerization of different synthetic monomers was employed for synthesizing hydrogels, including acrylic acid (AA), acrylamide (AAm), *N*‐isopropyl acrylamide (NIPAM), and PEG and polyvinyl alcohol (PVA).^[^ ^110^ ^]^ In contrast to natural hydrogels, synthetic hydrogels are typically fabricated from either low molecular weight monomers or high molecular weight polymers. These monomers can generate various types of hydrogels through diverse synthesis methods:^[^ ^111^ ^]^ 1) homopolymer hydrogels that consist of a crosslinked network of one hydrophilic unit; 2) copolymer hydrogels, which have two or more monomers, with at least one hydrophilic monomer; 3) interpenetrating polymer hydrogels, which are synthesized through a dual gelation process, whereby secondary monomers infiltrate the initial hydrogel, creating a secondary intermeshing network.

AA, an anionic monomer, has been widely developed to fabricate hydrogels with water retention and stretchability, making them valuable for applications in cosmetics and biomedicine. Wang et al. successfully utilized a commercial DLP 3D printer (405 nm UV light) to manufacture hydrogels using AA as the monomer, trimethylolpropane trimethacrylate as the crosslinker, and phenylbis(2,4,6‐trimethylbenzoyl)phosphine oxide as the photoinitiator.^[^ ^112^ ^]^ These hydrogels as soft actuators demonstrated significant deformation (up to 43°), rapid actuation speed (up to 1.08° s^−1^), and consistent bending performance across cycles under relatively low actuation voltage (4–6 V). Poly(acrylic acid) (PAA)‐based materials have also been applied in the antibacterial. Gratzel et al. reported PAA‐diblock copolymers, where some factors such as pH, electrolytes, salts, and polymer structure alterations impact the material's antimicrobial activity.^[^ ^113^ ^]^ Antimicrobial tests indicated that acid PAA copolymers had the highest antimicrobial efficacy on *E. coli* under slightly acidic conditions, attributed to the ion‐exchange effect.

NIPAM is a monomer commonly used to synthesize temperature‐responsive polymers, hydrogels, and other smart materials. NIPAM‐based hydrogels exhibit unique swelling and deswelling in response to changes in temperature, particularly around the lower critical solution temperature (LCST) of NIPAM, which is ≈32 °C. This responsiveness makes NIPAM‐based hydrogels useful in various biomedical and technological applications, including wound dressing, skin replacement, antibacterial applications, grippers, soft robots, smart valves, and bionics.^[^ ^114^ ^]^ As several studies have revealed the potential of hydrogels based on NIPAM produced through photopolymerization, NIPAM‐based hydrogels are fabricated using cutting‐edge light‐based 3D printing techniques, which is a departure from traditional manufacturing methods. Solis et al. pioneered the fabrication of poly(*N*‐isopropyl acrylamide) (PNIPAM) hydrogels utilizing a DLP 3D printer.^[^ ^115^ ^]^ The unique LCST feature of NIPAM determined the properties of the final products, encompassing swelling capacity, water retention, stress resilience, and deformation ability, where these properties can be finely tuned by adjusting various printing parameters, such as temperature, ranging from 5 to 15 °C. Furthermore, Mi et al. reported thermoresponsive NIPAM hydrogels with antimicrobial activity for wound dressing.^[^ ^116^ ^]^ These positively charged hydrogels were polymerized through reversible addition–fragmentation chain transfer polymerization, and then the antibacterial drug, salicylate, was absorbed within the structure of the hydrogel via electrostatic interaction. The betaine ester bond in the hydrogel is susceptible to hydrolysis, facilitating the release of the antibacterial drug. Experimental results demonstrated that salicylate was efficiently released within 12 h, leading to the complete inhibition of bacteria, specifically *E. coli* K12 growth. Can et al. also reported PNIPAM hydrogels by encapsulating antibacterial peptides (AMPs).^[^ ^117^ ^]^ This study efficiently encapsulated the AMP G(IIKK)3I‐NH_2_ into NIPAM solution at lower temperatures. Upon increasing the temperature above 33 °C, gelation occurred, leading to rapid drug release.

**Figure 5:**
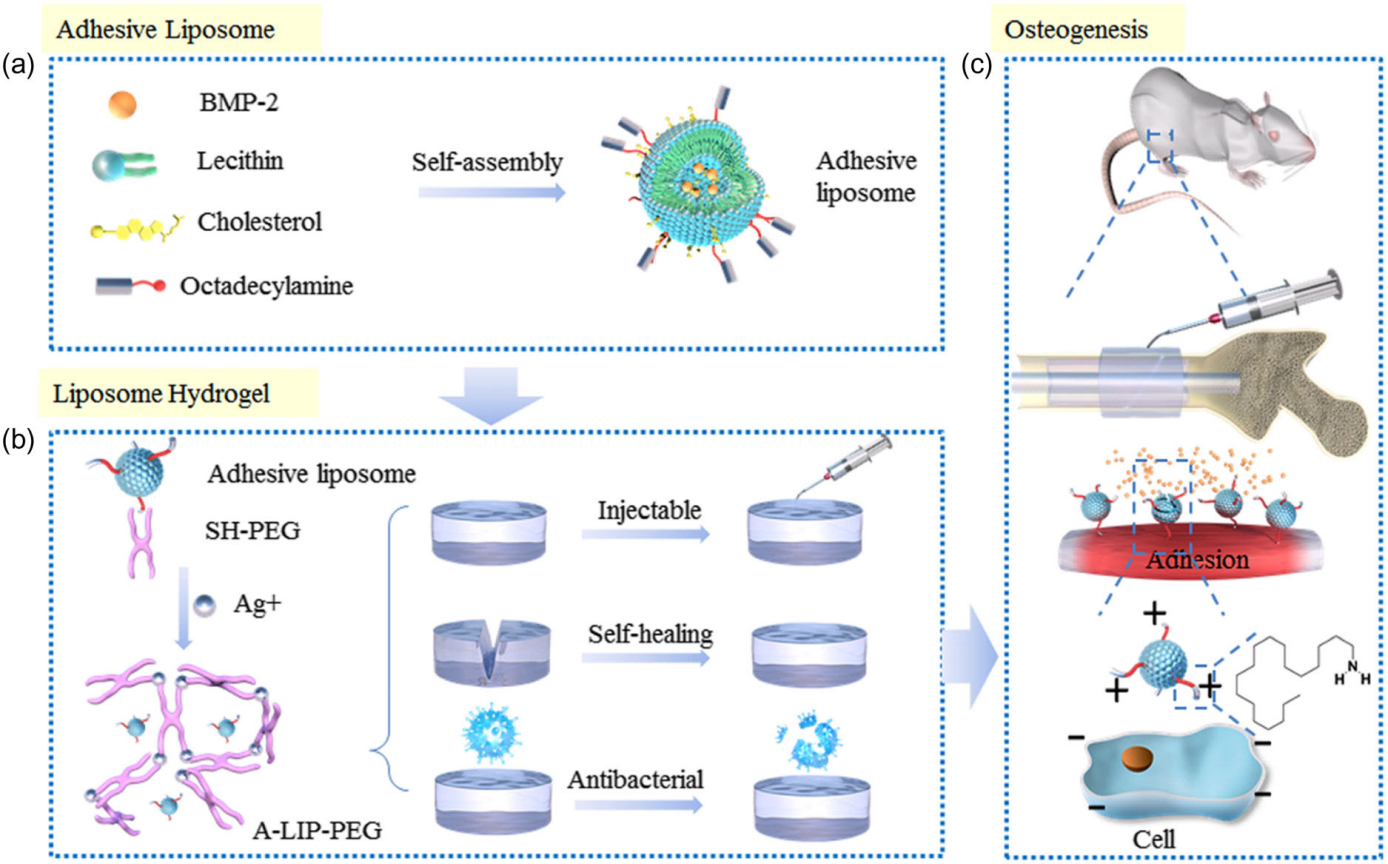
a) Schematic illustration of the formation of liposome hydrogel and Ag^+^. b) The formation of an adhesive, liposome‐laden, injectable, self‐healing, and antibacterial hydrogel via the Ag‐S coordination. c) The hydrogel was locally injected into an osteoporotic fracture and the bone marrow cavity for the release of drugs. Reproduced with permission.^[^ ^119^ ^]^ Copyright 2019, Springer Nature.

PEG, a hydrophilic polymer, is employed in making hydrogels for drug delivery, tissue engineering, and antibacterial materials,^[^ ^118^ ^]^ as PEG polymers possess various advantages, including nontoxic nature, minimal adverse reactions, degradability, and prolonged blood circulation. Liu et al. developed an injectable, self‐healing, antibacterial hydrogel by incorporating silver‐based liposomes into PEG materials (**Figure** **5**).^[^ ^119^ ^]^ Ag^+^ was utilized not only as a crosslinking agent but also as an antibacterial agent, achieving an inhibition rate of 89.7% ± 2.3% against *S. aureus*. Additionally, due to its tunable mechanical properties, PEG is often employed to enhance the mechanical properties of hydrogels with poor mechanical strength.^[^ ^120^ ^]^ Although the thermoresponsive behavior of NIPAM has garnered significant attention, simple NIPAM hydrogels often lack sufficient physical and mechanical properties, limiting further application. Sun et al. discovered that increasing the molecular weight of PEG in hydrogels enhances their mechanical properties by investigating the properties of different molecular weights of PEG in PNIPAM hydrogels.^[^ ^121^ ^]^ Poly(ethylene glycol) diacrylate (PEGDA), a derivative of PEG, is utilized to fabricate PEG‐based hydrogels, which are suitable carriers for drug delivery and other biomedical applications. More importantly, when exposed to light, PEGDA undergoes rapid photopolymerization to generate a crosslinked network. Therefore, PEGDA has been employed as an ink in light‐based 3D printing processes, facilitating the precise fabrication of hydrogels with excellent mechanical properties and biocompatibility.

PVA has been widely used to prepare high‐strength and roughness hydrogels. Hydroxyl groups of PVA facilitate strong intermolecular hydrogen bonding within the polymer network, resulting in enhanced mechanical strength of the hydrogel. Additionally, the abundant hydroxyl groups provide multiple sites for crosslinking with other molecules or polymers, further reinforcing the structure of the hydrogel. Recently, Ni et al. reported on a high‐speed underwater hydrogel robot based on PNIPAM and PVA. The incorporation of PVA not only enhances the mechanical properties of PNIPAM hydrogel but also improves its actuation angle.^[^ ^122^ ^]^ PVA has also found application in resin for light‐based 3D printing, where a DLP 3D printer was employed to fabricate PVA materials with tunable mechanical properties and cell compatibility.^[^ ^123^ ^]^ Overall, synthetic polymers have unique, superior, and exclusive physiochemical properties in contrast with natural polymers. Synthetic polymers are essential materials in light‐based 3D printing, enabling the fabrication of complex structures with tunable mechanical properties and biocompatibility, making them versatile for a wide range of applications in fields.

## 4 3D‐Printed Hydrogel Composites for Antibacterial Applications

Hydrogels themselves typically lack inherent antibacterial properties. Therefore, hydrogel composites composed of hydrogels combined with metal ions/metal‐based inorganic nanomaterials, antibiotics, and antibacterial polymers have been developed to improve their antibacterial efficacy. In addition, the incorporation of polymers or nanoparticles into hydrogel not only improves their mechanical properties, such as strength and stiffness, but also serves as crosslinkers to endow stimuli‐responsive behaviors in hydrogels.^[^ ^124^ ^]^ The following section will mainly focus on light‐based 3D‐printed hydrogel composites and their antibacterial properties.

### 4.1 Inorganic Nanomaterials

**Figure 6:**
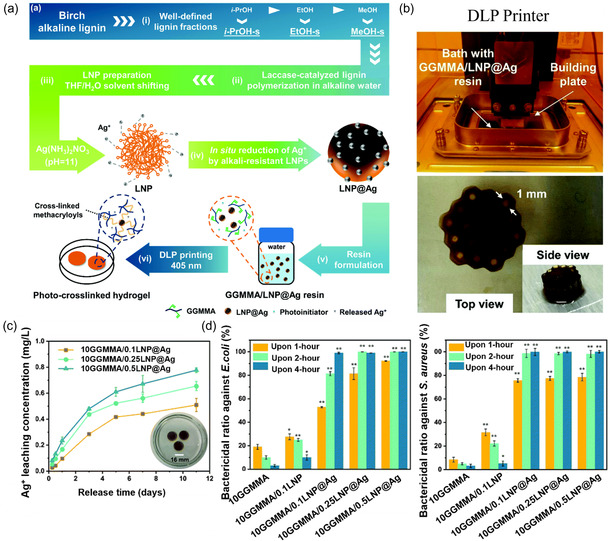
a) The design strategy for the sustainable resin and antimicrobial hydrogel involves the following steps: i) Preparation of laccasepolymerized lignin in alkaline aqueous media from well‐defined lignin fractions. ii) Fabrication of lignin nanoparticles. iii) In situ reduction of Ag^+^ on the surface of alkali‐resistant lignin nanoparticles. iv) Scheme of the molecular structure of photo crosslinkable resin. v) Scheme of the molecular structure of photo crosslinkable hydrogel. b) Fabrication of 3D objects by projection lithography. c) In vitro release profiles of Ag^+^ from hydrogels. d) Viability assays of *E. coli* and *S. aureus* on hydrogels. Reproduced with permission.^[^ ^34^ ^]^ Copyright 2022, Royal Society of Chemistry.

Inorganic nanoparticles have attracted tremendous attention in the biomaterial era due to their small size, large surface area,^[^ ^125^ ^]^ biodegradability,^[^ ^126^ ^]^ and intrinsic antibacterial properties.^[^ ^127^ ^]^ AgNPs, as one of the presentative metal‐based antibacterial agents, have been widely applied in antibacterial therapy.^[^ ^128^ ^]^ Various antibacterial mechanisms of AgNPs have been proposed, including disruption of the cell wall and cytoplasmic membrane, denaturation of ribosomes, interruption of adenosine triphosphate production, and production of reactive oxygen species (ROS).^[^ ^129^ ^]^ Particularly, the positive charge of the AgNPs allows for attachment to the bacterial membrane, creating structural changes in the cell membrane, promoting penetration into the cell, and ultimately leading to cell death.^[^ ^130^ ^]^ Furthermore, silver ions released from the nanoparticles can interact with thiol groups of proteins in bacteria, disrupting critical cell functions while generating ROS to further damage the bacteria.^[^ ^131^ ^]^ Integrating AgNPs into 3D printing techniques offers a valuable strategy to enhance the antibacterial properties of 3D‐printed hydrogels. These materials serve as a platform for the controlled and sustained release of silver ions, making AgNP composites more suitable for long‐term antibacterial therapy. Bergonzi and co‐workers innovatively developed 3D‐printed hydrogels incorporating AgNPs and alginate, enhancing antimicrobial properties.^[^ ^132^ ^]^ This novel approach demonstrated heightened effectiveness against pathogens and exhibited promising reductions in the risk of infections and toxicity. Experimental results found that *S. aureus* and *P. aeruginosa* growth was inhibited by ALG/CNC‐AgNPs growth in higher inhibition zones with concentrations of 10 and 100 μg mL^−1^. DLP technology has been reported to fabricate antimicrobial hydrogel composites by utilizing methacrylate *O‐*acetyl‐galactoglucomannan (GGMA) as the photopolymer, AgNPs as the antimicrobial agent, and lignin nanoparticles (LNPs).^[^ ^133^ ^]^ The LNPs’ joint action of antioxidant activity and UV shielding allows in situ reduction and stabilization of metal‐based nanoparticles. It was found that GGMMA/LNP@Ag hydrogel exhibited consistent release of Ag^+^ to the surrounding environment, resulting in a high antibacterial activity on *E. coli* and *S. aureus*. Wang et al. reported the DLP 3D printing of antimicrobial hydrogel embedded with AgNPs by using methacrylate‐substituted GGMA‐based resin. It was found that 10% GGMA with 0.5% AgNPs had the highest bactericidal ratio against both *E. coli* and *S. aureus* (**Figure** **6**).^[^ ^34^ ^]^ Furthermore, AgNPs have been conjugated with PAAm/hydroxypropyl methylcellulose (HPMC) and synergized with 3D printing for wound healing.^[^ ^134^ ^]^ The hydrogel was shown to have sufficient biocompatibility and biosafety on L929 fibroblast cell lines, indicating a low dose of AgNPs leaking from the crosslinked networks. Additionally, the AgNP‐PAAm/HPMC hydrogel composite was shown to have high levels of antibacterial effects on *E. coli and S. aureus*, achieving efficiencies of up to 86. 5 ± 3.2% and 75.8 ± 2.7%, respectively. Animal experiments showed that 3D‐printed hydrogel composite had increased wound healing in vivo mouse models, leading to faster wound closure with smoother surfaces and reduced scarring.

Copper also has gained popularity as a natural antibacterial agent. There are various antibacterial mechanisms. One widely accepted mechanism is the generation of ROS in the blasting reaction taking place within phagocytes.^[^ ^135^ ^]^ As with many metal‐based nanoparticles, the leached metal ions from CuNPs are positively charged and prone to interact with the surfaces of Gram‐positive and Gram‐negative bacteria. Additionally, copper ions from nanoparticles can interact with phosphorus and sulfur‐containing biomolecules, such as DNA and proteins, within bacterial cells.^[^ ^136^ ^]^ These interactions can lead to structural distortions within the bacteria, ultimately resulting in bacterial cell death. Mrialles‐Comins and co‐workers have developed a series of 3D printable polymerizable ionic liquids for DLP printing. In this study, the researchers prepared PEGDA‐based ink with 2 wt% copper dinitrate to fabricate materials using a DLP printer.^[^ ^137^ ^]^ It was found the addition of CuNPs in 3D‐printed materials boosts the antibacterial activity against *Staphylococcus epidermidis* (98%) and a significant inhibition zone with ≈4.5 mm wide was measured. Notably, they proposed a novel approach to synthesize copper materials in situ within the 3D‐printed films, simplifying the fabrication of antibacterial materials through light‐assisted 3D printing.

**Figure 7:**
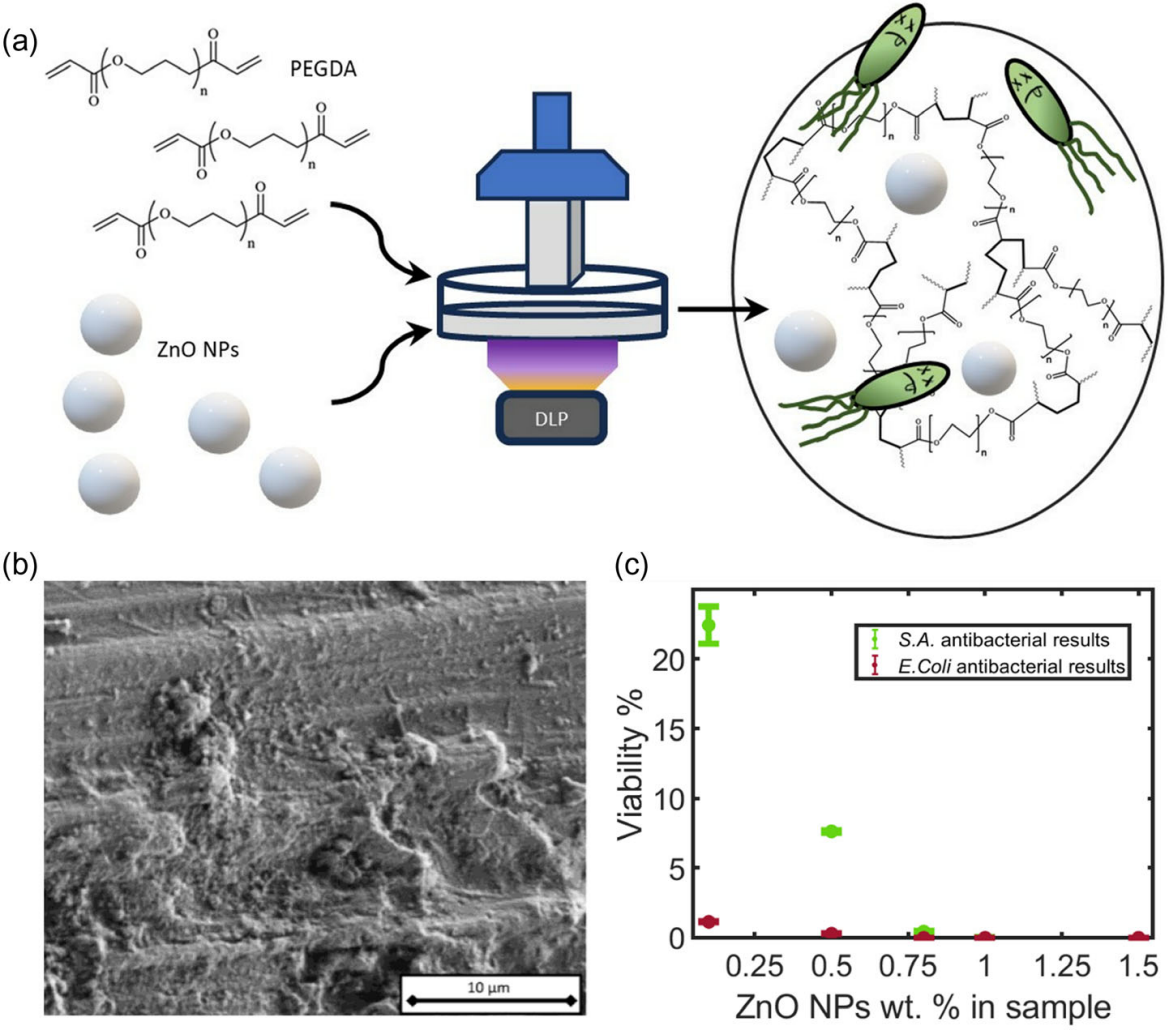
a) Schematics depict DLP printed PEGDA hydrogels containing ZnO NPs for antibacterial application; b) SEM images of DLP printed samples containing 1.5 wt% ZnO NPs; and c) *E. coli* and *S. aureus* viability percentage for different ZnO NP wt% in UV‐cured samples. Reproduced with permission.^[^ ^138^ ^]^ Copyright 2023, MDPI.

Zinc oxide nanoparticles (ZnO NPs), another metal oxide nanoparticle, have also been reported to serve as a 3D‐printed antibacterial agent. Mandler and co‐workers developed a 3D printable resin consisting of PEGDA and ZnO NPs to fabricate materials with similar mechanical properties to human cartilage.^[^ ^138^ ^]^ In this study, the ZnO NPs embedding matrix showed potent antibacterial activity against *E. coli* and *S. aureus*. The amount of ZnO NPs embedded in the matrix has only a minor effect on the mechanical properties but achieves strong antibacterial properties in the case of 1.5 wt% ZnO NPs (**Figure** **7**). Titanium dioxide nanoparticles have a large spectrum of activity against microorganisms, including Gram‐negative and Gram‐positive bacteria and fungi. Titania–polymer nanocomposites are environmentally friendly and exert a noncontact biocidal action.^[^ ^139^ ^]^ Cristache and co‐workers reported that the TiO_2_ was subjected to DLP 3D printing for denture manufacturing, exhibiting superior antibacterial efficacy. The prepared PMMA–TiO_2_ nanocomposite, containing 0.4%, 1%, and 2.5% TiO_2_ nanoparticles, inhibited the growth of the *Candida scotti* strain under standard conditions.^[^ ^140^ ^]^

Gold nanoparticles (AuNPs) exhibit adjustable optical and electronic features, making them indispensable in various applications, including photovoltaics, sensors, diagnostic imaging, and drug delivery.^[^ ^141^ ^]^ The critical property of AuNPs lies in their robust photothermal effects, enabling them to convert absorbed near‐infrared (NIR) efficiently light energy into heat. This feature positions AuNPs as a promising antibacterial material for ablating bacteria.^[^ ^142^ ^]^ For instance, when exposed to NIR light (808 nm) for 10 min, the temperature of a hybrid comprising AuNPs and PEG increased significantly from 23 to 55 °C.^[^ ^143^ ^]^ The in vivo photothermal antibacterial properties were initially validated using a subcutaneous implantation animal model. Moreover, hydrogel incorporated with AuNPs offers new options for fabricating next‐generation antibacterial materials. Batool et al. have synthesized an AuNP‐doped hydrogel for antimicrobial and wound healing potential.^[^ ^144^ ^]^ The hydrogel nanoparticle composite was shown to be effective against *Bacillus simplex*, *Bacillus subtilis*, *P. aeruginosa*, *E. coli*, and *S. aureus*, with the highest zone of inhibition for *E. coli*, *B. subtilis*, and *P. aeruginosa* at 18, 17, and 16 mm, respectively. Results showed that wound‐healing mouse models had 90% wound closure in treatment groups with AuNPs compared to the control groups. Furthermore, light‐assisted 3D printing technology has the potential in creating hydrogel composites containing AuNPs for antibacterial applications. To date, only a few articles have reported on the application of AuNPs in light‐based 3D‐printed materials for the photothermal treatment of bacteria. This limited use is primarily due to the significant amount of nanoparticles required in 3D printing processes, and AuNPs are relatively more expensive compared to other types of nanoparticles.

**Figure 8:**
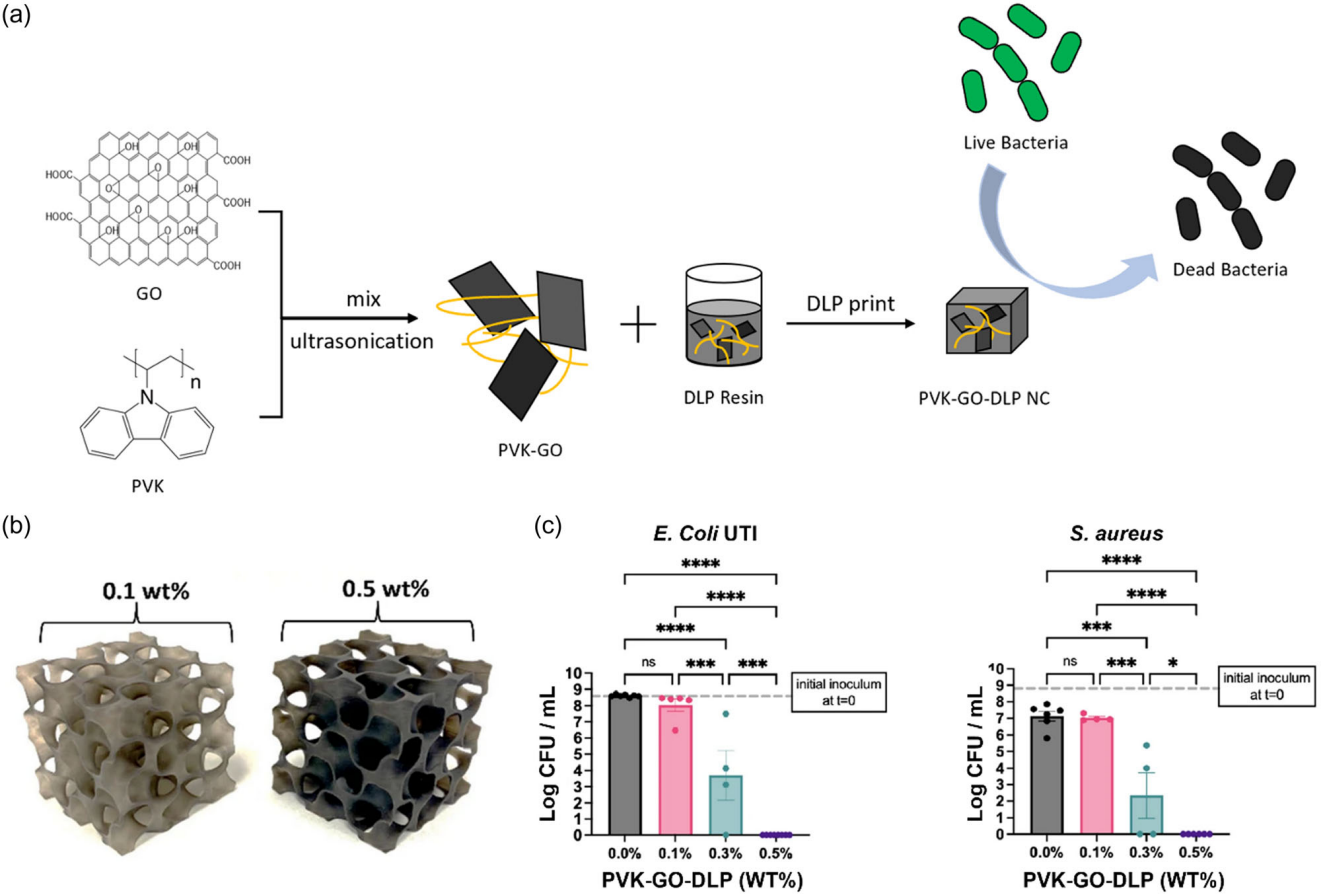
a) DLP printing of polymer‐based GO nanocomposite as an efficient antibacterial coating for surgical implants; b) DLP printed gyroid structures with 0.1 and 0.5 wt% nanocomposites; Quantitative culture of c) *E. coli* UTI and (b) *S. aureus* at 24 h postinoculation onto DLP printed materials. Reproduced with permission.^[^ ^33^ ^]^ Copyright 2023, Springer Nature.

Carbon‐based nanoparticles include graphene, carbon nanotubes, and carbon dots, which have been used for diagnostics, biosensing, imaging, and tissue engineering, owing to their unique properties such as high surface area and electrical conductivity.^[^ ^145^ ^]^ Another benefit of carbon‐based nanoparticles is their biocompatibility. Compared to metal‐based nanoparticles such as silver or copper, which tend to show cytotoxic effects,^[^ ^146^ ^]^ carbon‐based nanoparticles generally show lower toxicity.^[^ ^147^ ^]^ Furthermore, carbon‐based materials have emerged as a critical component in developing antibacterial applications. The mechanisms of carbon nanoparticles’ antibacterial action are diverse, primarily disrupting bacterial cell walls through interactions that induce cell death. The high surface area of the carbon‐based nanoparticles allows for efficient interactions with bacteria, which inhibit or neutralize bacteria.^[^ ^148^ ^]^ Furthermore, carbon nanoparticles possess photothermal capabilities under NIR light irradiation, enabling antibacterial therapy.^[^ ^149^ ^]^ Integrating carbon nanoparticles into 3D printing techniques offers a valuable approach to creating composites with enhanced benefits. Incorporating carbon nanoparticles can notably boost the mechanical properties of 3D‐printed hydrogels. For instance, graphene‐reinforced hydrogels demonstrate exceptional tensile strength, expanding the potential for this technology to be applied in medical scenarios requiring robust hydrogel strength.^[^ ^150^ ^]^ Carbon materials additionally offer photothermal or electrical properties when incorporated into 3D‐printed hydrogels. 3D‐printed composites containing carbon nanoparticles hold great potential for antibacterial therapy. Edaugal et al. operated DLP printing to fabricate polymer‐based GO nanocomposites as efficient antimicrobial materials (**Figure** **8**).^[^ ^33^ ^]^ They employed the noncovalent π–π interactions between poly(*N*‐vinyl carbazole) carbazole groups and GO to stabilize the dispersion of the nanofiller within the resin. The nanocomposites’ antibacterial efficacy was assessed against Gram‐negative *E. coli* and Gram‐positive *S. aureus*. The outcomes of this research exhibit the straightforward production of nanocomposites with exceptional antibacterial attributes using the DLP technique. These findings provide a basis for leveraging DLP as an efficient and economical manufacturing method for generating advanced antimicrobial materials, which could help reduce the incidence of bacterial‐related infections in biomedical devices, thus mitigating postoperative complications.

Metal–organic frameworks (MOFs), assembled by inorganic clusters and organic ligands through strong coordination bonds, exhibit high porosity, excellent stability, tunability, and advanced bio‐related features. These properties make MOFs attractive in bioimaging, drug delivery, biocatalysis, biosensing, and antibacterial applications.^[^ ^151^ ^]^ MOFs have emerged as an ideal material for diverse antibacterial applications due to their advantageous functions, including controlled or stimulated decomposition, strong interaction with bacterial membranes, formation of ROS under irradiation, and the capability for high loading and controlled release of other antibacterial agents.^[^ ^152^ ^]^ Integrating MOFs into 3D printing is an excellent way to improve the antibacterial ability of materials. For example, we have employed the SLA printing technique to PEGDA materials containing 2D porphyrinic MOFs.^[^ ^153^ ^]^ When exposed to light irradiation, 2D porphyrinic MOFs can transform oxygen into singlet oxygen, killing bacteria by inducing oxidative damage to cellular lipids, proteins, and DNA, disrupting essential cellular processes and leading to cell death. 3D‐printed materials have remarkable efficacy against both Gram‐positive and Gram‐negative bacteria at 98% and 93%, respectively, under an LED light (*λ*_max_ = 565 nm, 10 mW cm^−2^).

### 4.2 Antibiotics

**Figure 9:**
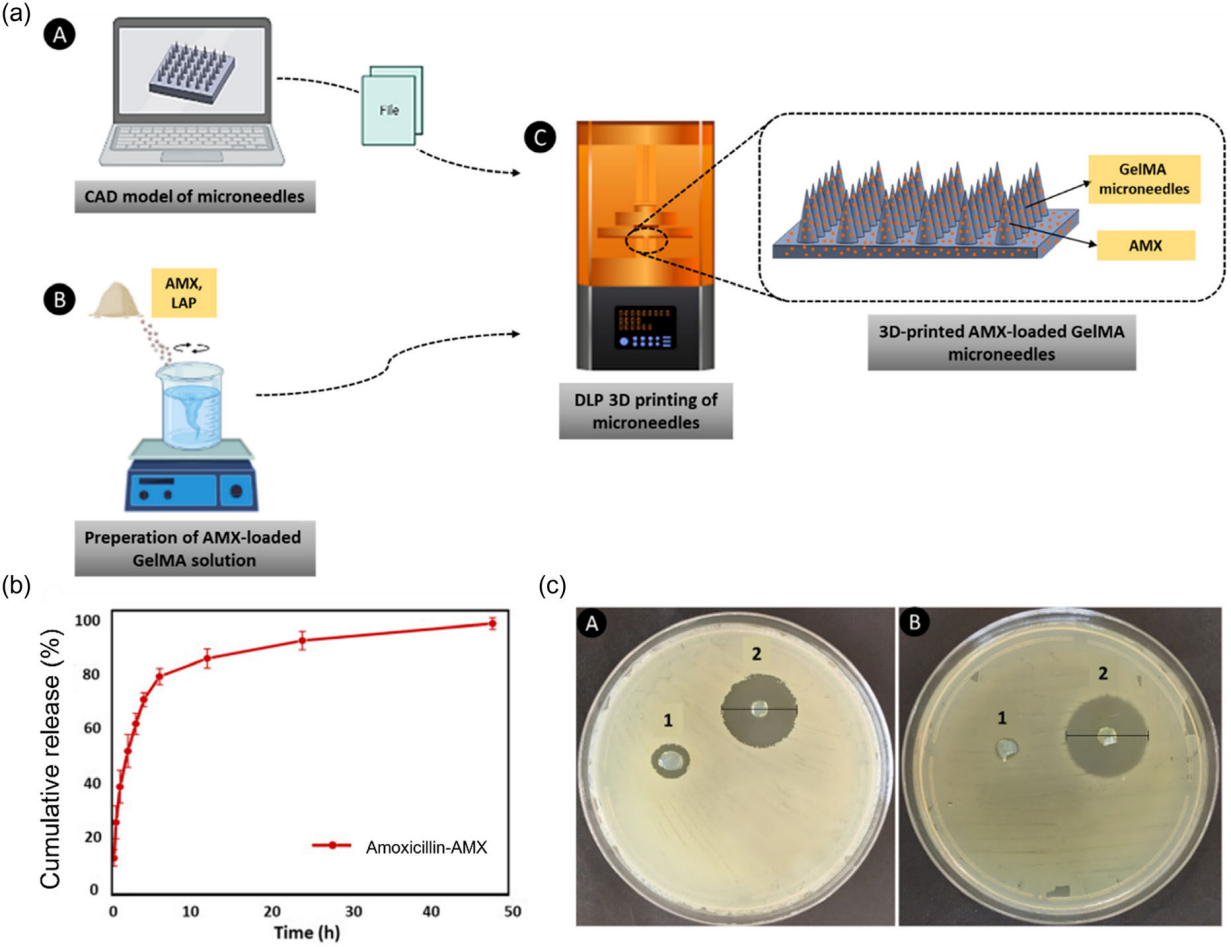
a) Schematic illustration of the design and DLP 3D printing of GelMA microneedles containing amoxicillin; b) drug release profile from the amoxicillin‐loaded GelMA microneedles; and c) antibacterial activities of the amoxicillin‐loaded GelMA microneedles: 1) GelMA microneedles and 2) AMX‐loaded GelMA microneedles. Reproduced with permission.^[^ ^157^ ^]^ Copyright 2023, Elsevier.

Antibiotics, since their inception with Alexander Fleming's discovery of penicillin in 1928, have significantly reduced mortality from bacterial diseases.^[^ ^154^ ^]^ There are different types of antibiotics, including penicillin, cephalosporins, quinolones, tetracyclines, macrolides, and aminoglycosides. Each antibiotic targets specific bacterial strains, necessitating tailored selection for optimal clinical efficacy.^[^ ^155^ ^]^ This personalized approach ensures effective treatment while mitigating antibiotic resistance. However, the indiscriminate use of antibiotics has led to antibiotic resistance, where bacteria can rapidly develop mechanisms to bypass the antibiotics’ mode of action. Developing hydrogel composites, which combine antibiotics, is an excellent way to solve this issue.^[^ ^5^ ^]^ Leveraging the high‐water content and customizable nature of hydrogels, antibiotics can be precisely delivered in a controlled manner. The goal is to selectively administer antibiotics, minimizing bacterial exposure and localized to specific areas of concern.^[^ ^5^ ^]^ Martinez‐Perez and co‐workers have employed 3D printing technology, utilizing GelMA and PLGA hydrogel as a platform, to develop a delivery system for rifampicin and vancomycin for antibiotic‐resistant bacteria.^[^ ^156^ ^]^ The amount of drug release and associated parameters were found to be dependent on the molecular weight of PLGA, with a lower molecular weight of PLGA associated with faster drug release. Erkus et al. used DLP printing techniques to manufacture a microneedle platform for transdermal drug delivery of amoxicillin (**Figure** **9**).^[^ ^157^ ^]^ These 3D‐printed microneedles exhibited rapid release of amoxicillin within the initial 6 h. Moreover, in vitro assessment revealed the effective antibacterial activity of the microneedles against both *E. coli* and *S. aureus*, with substantial zones of inhibition measuring 22 and 25 mm, respectively. This innovative approach demonstrates the potential for efficient transdermal delivery of antibiotics and effective treatment of bacterial infections.

### 4.3 Cationic Polymer‐Based Antibacterial Materials

In the last decades, there has been a growing interest in developing cationic polymers containing quaternary, phosphonium, and pyridinium cations as antimicrobials because of their low cost, broad spectrum, and antibacterial activity.^[^ ^158^ ^]^ In general, cationic polymers act antibacterial effects upon the negatively charged membrane of the bacteria through electrostatic interaction, followed by insertion of their hydrophobic segments into the lipid domains to kill the bacteria.^[^ ^159^ ^]^ However, these cationic polymers also inevitably damage human cell membranes during antimicrobial therapy, causing toxicity.^[^ ^160^ ^]^ Developing cationic polymer‐based materials with low toxicity is an urgent priority. Ting et al. developed a bilayer asymmetric hydrogel to replicate the skin's epidermis and dermis gradient structure, utilizing a combination of electrostatic spinning and 3D printing techniques. The hydrogel exhibited surface hydrophilicity, hydrophobicity, porosity, mechanical strength, and antibacterial capabilities. The inner layer, composed of sodium alginate/PVA/chitosan quaternary ammonium salt, demonstrated significant antibacterial activity against *S. aureus*, with an inhibition zone measuring 1.61 ± 0.35 cm.^[^ ^161^ ^]^

In addition, AMPs, one of the antibacterial cationic polymers, exhibit sensitivity toward a wide range of bacteria, fungi, and viruses. Like cationic polymers and some metal nanoparticles, AMPs can bind to bacterial cell membranes, inducing the formation of pores on the surface of membranes, ultimately leading to bacterial death. For example, Maleki and co‐workers printed hybrid scaffolds containing AMPs for repairing bone defects and antibacterial.^[^ ^162^ ^]^ Experiments conclusively confirmed that the 3D‐printed material exhibited a significant zone of bacterial inhibition against Gram‐positive bacteria, which was significantly better than the antimicrobial effect against Gram‐negative bacteria. This is because of the presence of lipopolysaccharides in the outer membrane of Gram‐negative bacteria, which functions as a protective shield by impeding the access of CM‐RGD to the inner cytoplasmic membrane.

## 5 Conclusion

Light‐assisted 3D printing is a revolutionary technique that provides the possibility of fabricating advanced antibacterial hydrogels for more diverse and intricate clinical scenarios. There are several advantages, including a wide selection of photopolymer materials, the creation of highly precise, customized antibacterial materials crafted to meet specific applications or requirements, as well as the ability to exert precise control over the distribution and release of antibacterial agents within 3D‐printed hydrogels. In this review, we summarized the unique properties, antibacterial ability, and potential applications of natural and synthetic hydrogels fabricated by light‐assisted 3D printing. Moreover, we discussed current approaches to improve and prolong the antimicrobial efficacy of 3D‐printed hydrogels through the combination of potent antibacterial agents. The objective of our review is to provide researchers with an up‐to‐date understanding of the advancements in light‐assisted 3D‐printed hydrogels.

It is noteworthy that there are issues in both light‐assisted 3D printing and antibacterial hydrogels that require attention in the future. 1) The main light source of light‐assisted 3D printing (such as SLA and DLP) is UV light/beams, which inevitably causes damage to living cells; 2) A standard 3D printing antibacterial resin entails a complex formulation, which is composed of photoinitiators, monomers/crosslinkers, cells, and other functional compounds. The development of ideal resins that balance optimal biocompatibility and potent antibacterial properties remains a critical pursuit; and 3) The introduction of light‐assisted 3D printing has not effectively addressed the issues posed by the weak mechanical performance and fragility of hydrogels, thus restricting their specific applications, like bone implants and vascular tissue engineering.

Innovation in 3D printing techniques and hydrogel formulations is expected to tackle current obstacles, broadening scenarios of antibacterial materials. For instance, researchers have developed a novel visible light‐assisted 3D printer, which is able to mitigate the potential side effects of UV light/beams in biomedicine. Furthermore, incorporating artificial intelligence and machine learning algorithms into the field of 3D‐printed hydrogels can revolutionize the printing parameters and formulation design. Artificial intelligence and machine learning can identify intricate patterns and relationships, facilitating the optimization of 3D printing parameters for the fabrication of antibacterial hydrogels. This data‐driven approach accelerates the process of developing and designing new resins while maintaining precision and efficacy. Artificial intelligence and machine learning also predict hydrogel behaviors, aiding in customizing formulations tailored to specific applications. Therefore, collaborative interdisciplinary initiatives involving materials scientists, bioengineers, computer scientists, and healthcare practitioners will propel advancements in this field and address the multifaceted challenges associated with bacterial infections.

## Conflict of Interest

The authors declare no conflict of interest.
